# Lentiviral CRISPR-guided RNA library screening identified Adam17 as an upstream negative regulator of Procr in mammary epithelium

**DOI:** 10.1186/s12896-021-00703-9

**Published:** 2021-07-19

**Authors:** Ting Wu, Yinghua Wang, Tianxiong Xiao, Yirui Ai, Jinsong Li, Yi Arial Zeng, Qing Cissy Yu

**Affiliations:** 1 State Key Laboratory of Cell Biology, CAS Center for Excellence in Molecular Cell Science, Institute of Biochemistry and Cell Biology, Chinese Academy of Sciences, University of Chinese Academy of Sciences, Shanghai, 200031 China; 2 School of life Science, Hangzhou Institute for Advanced Study, University of Chinese Academy of Sciences, Chinese Academy of Sciences, Shanghai, China

**Keywords:** CRISPR-Cas9, gRNA library, Procr, Adam17

## Abstract

**Background:**

Protein C receptor (Procr) has recently been shown to mark resident adult stem cells in the mammary gland, vascular system, and pancreatic islets. More so, high Procr expression was also detected and used as indicator for subsets of triple-negative breast cancers (TNBCs). Previous study has revealed Procr as a target of Wnt/β-catenin signaling; however, direct upstream regulatory mechanism of Procr remains unknown. To comprehend the molecular role of Procr during physiology and pathology, elucidating the upstream effectors of Procr is necessary. Here, we provide a system for screening negative regulators of Procr, which could be adapted for broad molecular analysis on membrane proteins.

**Results:**

We established a screening system which combines CRISPR-Cas9 guided gene disruption with fluorescence activated cell sorting technique (FACS). CommaDβ (murine epithelial cells line) was used for the initial Procr upstream effector screening using lentiviral CRISPR-gRNA library. Shortlisted genes were further validated through individual lentiviral gRNA infection followed by Procr expression evaluation. Adam17 was identified as a specific negative inhibitor of Procr expression. In addition, MDA-MB-231 cells and Hs578T cells (human breast cancer cell lines) were used to verify the conserved regulation of ADAM17 over PROCR expression.

**Conclusion:**

We established an efficient CRISPR-Cas9/FACS screening system, which identifies the regulators of membrane proteins. Through this system, we identified Adam17 as the negative regulator of Procr membrane expression both in mammary epithelial cells and breast cancer cells.

**Supplementary Information:**

The online version contains supplementary material available at 10.1186/s12896-021-00703-9.

## Background

Procr, also known as endothelial protein C receptor (EPCR), is a single-pass transmembrane protein with reported function in anticoagulation, inflammation and hematopoiesis [1–5]. Our previous studies have identified Procr as an adult stem cell marker of multiple tissues, such as the mammary gland epithelium, blood vasculature, ovary epithelium and pancreatic islets [6–9]. Moreover, almost half of aggressive triple negative breast cancer (TNBC) cases have been shown to harbor robust PROCR expression [10], indicating that PROCR could be a useful biomarker as well as a potential treatment target. Previous study has revealed Procr as a target of Wnt/β-catenin signaling [6]; however, direct upstream regulatory mechanism of Procr remains unknown. To comprehend the molecular role of Procr during physiology and pathology, elucidating the upstream effectors of Procr is necessary.

Genome-wide loss-of-function screening is a powerful approach used to discover modulating genes and pathways underlie biological processes. CRISPR-Cas9 system mediated genome editing can effectively inactivate genes, which is frequently used in loss-of-function screening for systemic genetic analysis [11, 12]. The development of various guide RNA (gRNA) libraries further aids in the efficiency, whereby cells were transfected/infected with pre-determined sets of gRNA for large-scale functional screens. To select for cells with interested phenotypic output, consequent enrichment procedures are required, including drug selection or antibiotic challenging [11–13]. These methods help to remove “noise” cells and isolate target cells with the gRNAs that resulted in the desired phenotype.

In this study, we established a screening system that combines CRISPR-Cas9 mediated gene disruption and FACS-based cell surface protein expression analysis. Using this approach, we identified Adam17 as a negative upstream regulator of Procr protein expression. This regulation was also verified in TNBC cells. Considering inhibition of PROCR suppresses tumor growth from transplanted PROCR^+^ breast cancer cells [10], ADAM17 could provide a new potential therapeutic target for PROCR^+^ TNBCs.

## Results

### Lentiviral CRISPR-guide RNA library infection

**Fig. 1:**
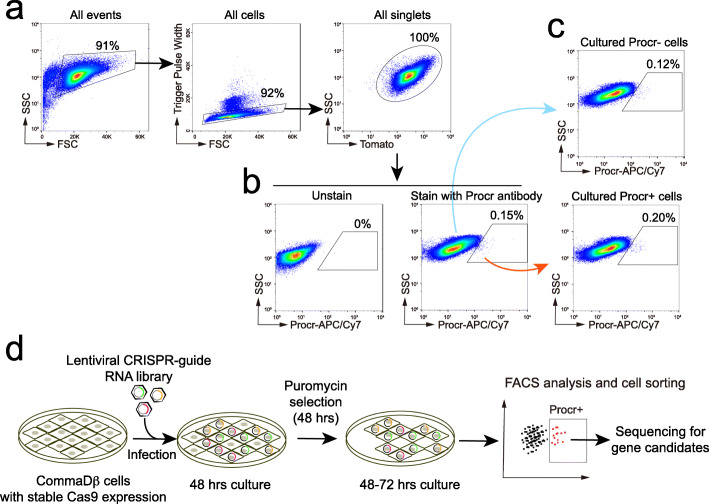
Lentiviral CRISPR-guide RNA library infection. **a** FACS analysis gating strategies. **b** FACS analysis of Procr expression in CommaDβ cells. Out of total CommaDβ cells, 0.15% of cells have endogenous Procr expression (Procr^+^). **c** Procr^−^ or Procr^+^ CommaDβ cells were isolated and separately cultured for 10 days. FACS analysis of cultured cells showed similar Procr^+^ cell fractions regardless of their culture origin. **d** CommaDβ cells with stable Cas9 expression were infected with lentiviral CRISPR-guide RNA library. After 2 days, cells were treated with puromycin for 48 h for positive selection. After puromycin selection, cells were further cultured for 2–3 days in fresh medium before FACS analysis for Procr expression

To enable CRISPR-gRNA mediated screening, we generated stable -Cas9-expressing CommaDβ cells, a mouse mammary epithelial cell line. We first examined the endogenous expression of Procr in CommaDβ cells by staining with mouse Procr antibody followed by Streptavidin-APC/Cy7 conjugated secondary antibody (Fig. 1a). FACS analysis indicates that CommaDβ cells have low endogenous Procr expression (0.15%, Fig. 1b). We isolated Procr^+^ and Procr^−^ CommaDβ cells and found that cultured progeny of either population showed similar proportion of Procr expression after passage (Fig. 1c). This suggests that Procr expression is dynamically regulated and can be induced. To prepare for functional screen, we used Cas9 PB transposon system to generate CommaDβ cells with stable and sustained Cas9 expression. Single cells were cultured separately and clonal expanded and colonies with uniform Cas9 expression were selected for screening.

We next infected Cas9-stable expressing cells with a lentiviral CRISPR-gRNA library [11]. In this mouse genome-wide lentiviral gRNA library, a total of 19,150 mouse protein coding genes are targeted. Only the overlapping regions were chosen for genes with multiple CDS transcripts. The sequences of each genomic interval were retrieved and used to identify all sequences comprising 5′-N19NGG-3′. Filtering was performed as follows: First, sites with more than 1 perfect hit in any of the Ensembl exons were removed. Second, off-target sites of each candidate gRNA were examined with the following two options (i) N12NGG without any mismatches and (ii) N20NGG with up to three mismatches. Third, gRNAs that are positioned at least 100 bp away from the translation initiation site and in the first half of coding sequences were collected. This library contains 87,897 gRNAs covering 94.3% of genes with 2–5 gRNAs chosen for each gene, prioritizing gRNAs with fewer predicted off-target sites. After infection, cells were allowed to recover for 2 days, followed by 48 h Puromycin treatment for selecting positively infected cells. Cells were further culture for 2–3 days before FACS analysis for Procr expression. All the Procr^+^ cells were then FACS isolated and expanded in culture (Fig. 1d).

### Screening for upstream regulators of Procr by lentiviral CRISPR-gRNA library

**Fig. 2:**
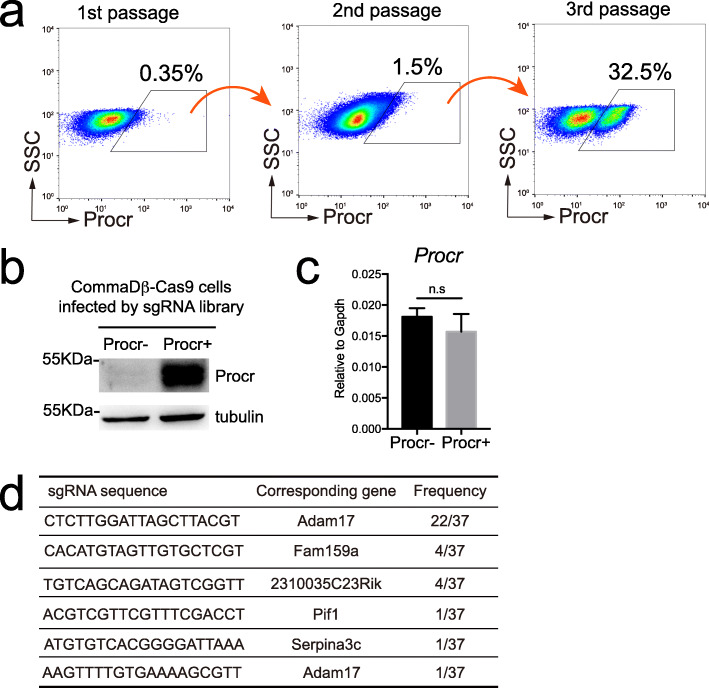
CRISPR-gRNA library screening for upstream regulators of Procr. **a** Procr^+^ cells were sorted by FACS and expanded by serial passage. In third passage, a population with high Procr expression emerged. **b** Western blot analysis confirming high Procr protein level in FACS isolated Procr^+^ cells. **c** RT-qPCR analysis showing similar Procr mRNA transcription level between FACS isolated Procr^−^ and Procr^+^ cells. **d** The genomic DNA of FACS isolated Procr^+^ cells were extracted and gRNAs which were integrated in the genome were sequenced. Five regulatory gene candidates were identified

After lentiviral CRISPR-gRNA library was introduced into Cas9-stable expressing CommaDβ cells, approximately 20,000 Procr^+^ cells were FACS isolated and serially expanded. In each passage, Procr^+^ fractions were enriched by FACS sorting and subsequently cultured. Sequential FACS analysis on each passage showed a slight increase in Procr^+^ cells in the second passage, and by the third passage, a population with high Procr expressing emerged (Fig. 2a). We then performed western blot and qPCR analysis. Western blot results confirmed the increase in Procr protein level (Fig. 2b), however, the Procr mRNA levels remain unchanged (Fig. 2c). Therefore, the candidate genes, which were disrupted, likely exert their regulation over Procr translation or protein stability, instead of its transcription.

To identify the key effectors responsible for changes in Procr protein level, we isolated Procr^+^ cells from the third passage culture and performed genomic DNA extraction. The sequences containing sgRNA element were then amplified by PCR and subsequently ligated into pMD-19 T vectors. A total of 37 clones were picked for sanger sequencing to identify the targeted gRNAs. 22 out 37 were Adam17 gRNAs (there are two variant Adam17 gRNAs detected in the mutant Procr^+^ cells), gRNA sequences of Fam159a, 2310035C23Rik, Pif1, Serpina3c were also detected in the mutant Procr^+^ cells (Fig. 2d).

### Functional validation of the potential candidates

**Fig. 3:**
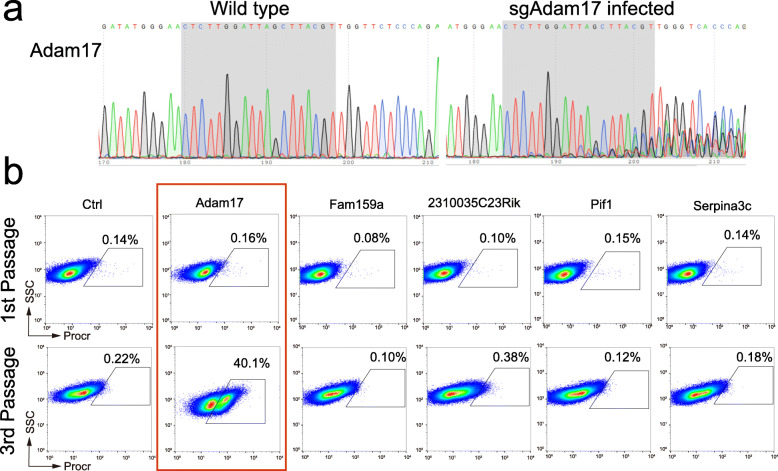
Functional validation of potential candidate genes. **a** Wild-type and Adam17-sgRNA infected cells were sequenced to validate the mutation in *Adam17* gene. **b** CommaDβ cells were infected with individual lentiviral gRNA of Ctrl, Adam17, Fam159a, 2310035C23Rik, Pif1, Serpina3c. In the third passage, only cells with Adam17 gRNA had a significant Procr^+^ population

To verify the target genes that caused the changes in Procr protein level, we constructed single guide RNA (sgRNA) for each of the 5 candidates and separately infected CommaDβ cells following the same procedures as in Fig. 1d. Both wild-type and sgRNA infected cells were DNA-extracted for sequencing. Sequencing of sgRNA-targeting regions verified targeted mutation of each candidate genes (Fig. 3a, Fig. S2). Infected cells were cultured for 3 passages and then FACS analyzed for Procr surface expression. Out of the 5 candidates, only Adam17 disruption lead to the increase in Procr^+^ cell proportion, while the disruption of other four genes or control gRNA didn’t (Fig. 3b). Adam17 is a shedding protease that can cleave variety of substrates, such as interleukin-6 receptor (IL-6R), the pro-inflammatory cytokine tumor necrosis factor alpha (TNFα) and most ligands of the epidermal growth factor receptor (EGFR) [14–21]. Previous report has shown that ADAM17 could cleave PROCR in endothelial cells [22]. Here, our results showed Adam17 also fine-tunes Procr protein level in mammary epithelial cells.

### Knockdown of ADAM17 increases PROCR level in TNBC cell lines

**Fig. 4:**
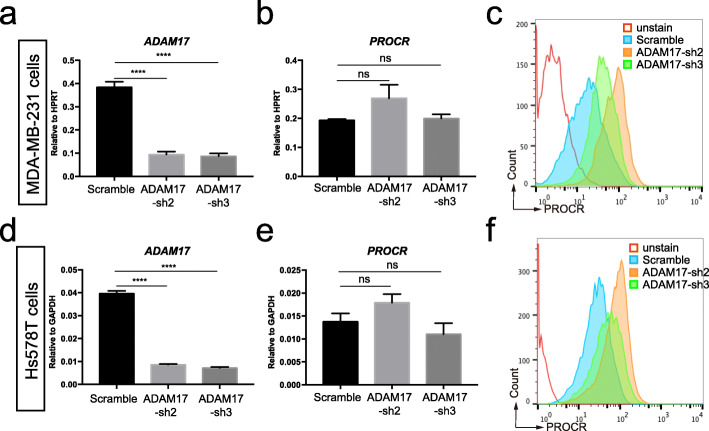
Knockdown of *ADAM17* increases PROCR level in TNBC cell lines. **a** Validation of *ADAM17* shRNAs knockdown efficiency in MDA-MB-231 cells by RT-qPCR. **b** RT-qPCR results showing *PROCR* mRNA remain unchanged in MDA-MB-231 cells after knockdown of *ADAM17*. **c** Knockdown of *ADAM17* in MDA-MB-231 cells resulted in increased protein levels of membrane expressing PROCR. **d** Validation of *ADAM17* shRNAs knockdown efficiency in Hs578T cells by RT-qPCR. **e** RT-qPCR results showing PROCR mRNA remain unchanged in Hs578T cells after knockdown of *ADAM17*. **f** Knockdown of *ADAM17* in Hs578T cells resulted in increased protein levels of membrane expressing PROCR. a-b, d-e, Data are presented as mean ± S.E.M. n.s, not significant. ****P < 0.0001

Our lab has previously demonstrated that PROCR is highly expressed in about half of the TNBC incidences. Therefore, we want to find out whether ADAM17 also regulate PROCR expression in human breast cancer cells. To this end, we used MDA-MB-231 and Hs578T cells (both are TNBC cell lines) and generated shRNAs of ADAM17. Knockdown efficiency of sh*ADAM17*s validated in both cell lines (Fig. 4a, d). Consistent with results from CommaDβ cells, knockdown of ADAM17 didn’t affect the mRNA levels of PROCR in neither MDA-MB-231 nor Hs578T cells (Fig. 4b, e), while PROCR protein levels were significantly increased as shown by FACS analysis (Fig. 4c, f). These results suggest that ADAM17 also functionally downregulated PROCR protein levels in TNBC cell lines.

## Discussion

In this study, we developed an efficient method to screen upstream effectors of target cell surface proteins, through combining CRISPR-Cas9 mediated gene disruption and FACS-based cell surface protein expression detection. Using this system, we screened and identified Adam17 as a negative regulator of Procr protein expression in both mammary epithelial cells and breast cancer cells, suggesting potential therapeutic significance.

Procr was demonstrated as an adult stem cells marker in multiple tissues. Moreover, High PROCR expression has been detected in almost half of the aggressive TNBC incidences [10]. Functional significance of PROCR was also emphasized in tumor transplantation models where the knockdown of *PROCR* in PDX tumor cells resulted in reduced tumor size and enhanced recipient survival rate [10]. These altogether suggest that elucidating the upstream regulation of PROCR, in particular, identifying the effectors that down-regulate PROCR, could hold clinical relevance. In line with this, we used CommaDβ, a mammary epithelium cell line that has low endogenous Procr expression (~ 0.2%), which enables sensitive detection in Procr expression changes. Through CRISPR-Cas9 mediated gene loss-of-function screen, we identified negative regulators of Procr, which yield the FACS detectable output of increased Procr protein expression. In the same ways, by using cells with high target surface protein expression, this method could be modified for the selection of expression enhancers.

In the mammary gland, previous study has demonstrated Procr as a Wnt pathway target gene [6], Procr expression upregulates in response to Wnt signaling activation. Reciprocally, we anticipated the silencing of Wnt inhibitors would result in augmented Procr expression. Surprisingly, none of the Wnt signaling related genes appeared in the candidature list. This is likely due to the low expression level of Wnt signaling activities in CommaDβ cells [23]. Silencing of the inhibitors of Wnt signaling didn’t lead to sufficient signaling pathway activation, hence, no change in Procr expression observed. It is also possible that our method of Sanger’s sequencing did not provide in depth coverage of affected genes, which could be improved by a comprehensive analysis of all the sgRNAs in the lentiviral library using high-throughput sequencing. To compensate for silenced signaling activities during in vitro cell line generation and passages, multiple cell lines may be used to broaden the coverage of screened candidate effectors.

ADAM17 belongs to the ADAM protein family and was the first disintegrin metalloprotease to be molecularly characterized. Studies have described the role of ADAM17 as an important regulatory hub in development, immunity and cancers [15, 24–26]. ADAM17 functions as a shedding protease that can cleave variety of substrates, including endothelial PROCR and EGFR ligands [5, 18, 20, 22]. In endothelial cells, the proteolytic cleavage of PROCR generates a soluble ectodomain of PROCR, called soluble PROCR (sPROCR) which exerts anticoagulant activity through the inhibition of activated protein C [22, 27]. In our study, we identified Adam17 as the major regulator of Procr protein level in mammary epithelial cells as well as in MDA-MB-231 and Hs578T breast cancer cells. Whether this ADAM17 mediated protein-shedding process also produce sPROCR in mammary epithelial cells remains unknown. Since PROCR knockdown could significantly ameliorate tumor growth in transplantation [10], the therapeutic potential of ADAM17 in TNBC is worth investigating.

## Conclusion

The established CRISPR-Cas9/FACS screening system efficiently identified the regulators of membrane proteins and we identified Adam17 was the negative regulator of Procr both in mammary epithelial cells and breast cancer cells.

## Methods

### Cell culture

CommaDβ, MDA-MB-231 and Hs578T cell lines were obtained from the Committee on Type Culture Collection of Chinese Academy of Sciences (Shanghai, China). All cells were maintained in DMEM medium (Gibco, cat#C11995500), with 10% Fetal bovine serum (FBS; Hyclone, cat#SH30896.03) and 1% penicillin-streptomycin at 37 °C with 5% CO^2^. CommaDβ cells were passaged every 3 days or till reach confluency. Before gRNA lentivirus infection, the transducing units of the lentivirus were detected before infection. And the ratio of virus to cell is 1:1 to make sure the infected cells with low MOI. After lentiviral infection, cells were cultured for 48 h before puromycin selection. Puromycin was supplemented into the cell culture for 48 h followed by a change of fresh medium. Cells were culture for another 48–72 h to allow recovery and expansion.

### Generation of Cas9 stable expression cell line

CommaDβ cells were transfected with the plasmid PiggyBac-Cas9 carrying an RFP reporter gene and plasmid PiggyBac transposase using lipofectamine 3000 following the manufacture instruction. Three days after transfection, single RFP^+^ cells were sorted into 96-well plate by FACS. Cells with high RFP signals were cultured and expanded.

### CRISPR-gRNA library construction

We employed a characterized mouse lentiviral gRNA library that contains 87,897 gRNAs targeting 19,150 mouse protein-coding genes, covering 94.3% of genes with at least two gRNAs per gene [11]. The lenti-sgRNA library plasmids were purchased from Addgene (Pooled Library #50947). When genes have multiple CDS transcripts, only the overlapping regions were chosen. The sequences of each genomic interval were retrieved and used to identify all sequences comprising 5′-N19NGG-3′. Filtering was performed as follows: First, sites with more than 1 perfect hit in any of the Ensembl exons were removed. Second, off-target sites of each candidate gRNA were examined with the following two options (i) N12NGG without any mismatches and (ii) N20NGG with up to three mismatches. Third, gRNAs that are positioned at least 100 bp away from the translation initiation site and in the first half of coding sequences were collected. Finally, up to 5 gRNAs were chosen for each gene, prioritizing gRNAs with fewer predicted off-target sites.

For preparation of the lentivirus, the HEK293T cells in a 10-cm dish were transfected with 3 μg of lenti-sgRNA library plasmids and 9 μg of ViraPower Lentiviral Packaging Mix (Invitrogen) by Lipofectamine 2000 Reagent (Invitrogen) in accordance with the manual. The supernatant was harvested 72 h after transfection, concentrated with Lenti-Concentin virus precipitation solution (SBI), and then stored at − 80 °C. In order to determine the virus volumes for achieving an MOI of 0.3 to ensure that most cells receive single copy of the lentiviral vector, HEK293T in 24-well plates were incubated with different volume of lentivirus.

### Guide-RNA sequencing

200,000 mutant Procr^+^ cells were harvested for DNA extraction. The gRNA-encoding regions were then amplified by PCR. The PCR fragments were ligated into pMD-19 T vectors. A total of 37 clones were picked for sanger sequencing to identify the targeted gRNAs.

### Flow cytometry and antibodies

Cultured CommaDβ-Cas9 or MDA-MB-231 cells were washed with PBS (Gibco, cat#00095) and treated for 5 mins with 0.25% trypsin (Gibco, cat#25200) for cell harvest. Cells were centrifuged and suspended with 5% fetal bovine serum (FBS; Hyclone, cat#SH30896.03) in PBS. After incubation with primary antibodies for 20 mins on ice, the cells were washed by PBS, and resuspended in 5% FBS. Cells were incubated with secondary antibody when needed. After antibody incubation, cells were washed with PBS and filtered through 40 μm strainers to obtain single cell suspension before FACS analysis. The following antibodies in 1:200 dilutions were used: APC-anti-human PROCR (eBioscience, cat#17–2018-42), Biotin-anti-mouse Procr (eBioscience, cat#12–2012-82), Streptavidin-APC/Cy7 (Biolegend, cat#405208). All analysis and sorting were performed using a FACSJazz (Becton Dickinson). The purity of sorted population was routinely checked and ensured to be > 98%.

### Western blot and antibodies

Cells were FACS isolated or harvested from and lysed in SDS sample buffer. Proteins were separated by SDS-PAGE and blotted onto polyvinylidene fluoride membranes. Primary antibodies used in Western blot were rabbit anti-mouse Procr (1:200, generated in the lab), mouse anti-tubulin (15,000, Sigma, cat#T5168). Secondary antibodies used in Western blot were Goat anti rabbit-HRP (Cell Signaling Technology, cat#7074), goat anti mouse-HRP (Cell Signaling Technology, cat#7076).

### RNA isolation and real time-qPCR

Cells were lysed in RNAiso plus (Takara, cat#D9108A) and RNA isolation was performed following manufacturer’s instructions. Extracted RNA was reverse transcribed into cDNA using the Primerscript RT master kit (Takara, cat#RR036A). cDNA samples and primers were prepared with SYBR Green Mixture (Roche, cat#04913914001) and detected using Applied Biosystems Stepone Plus detection system. The results were processed by ΔΔCt algorithm. Primers used in qPCR analysis were as followed (5′-3′):

Mouse Gapdh-F, TGTGATGGGTGTGAACCACGAGAA.

Mouse Gapdh-R, CTGTGGTCATGAGCCCTTCCACAA.

Mouse Adam17-F, ACCACTTTGGTGCCTTTCGT.

Mouse Adam17-R, GTCGCAGACTGTAGATCCCTT.

Mouse Procr-F, GCATGTTGACGAAGTTTCTGCCG.

Mouse Procr-R, GCTTAGCAACGCCGTCCACTTG.

Human GAPDH-F, ACATCGCTCAGACACCATG.

Human GAPDH-R, TGTAGTTGAGGTCAATGAAGGG.

Human ADAM17-F, GTGGATGGTAAAAACGAAAGCG.

Human ADAM17-R, GGCTAGAACCCTAGAGTCAGG.

Human PROCR-F, GCATGTTGACAACATTGCTGCCG.

Human PROCR-R, GCTTAACATCGCCGTCCACCTG.

### ADAM17-shRNA lentivirus package and infection

ADAM17-shRNAs were cloned into pLKO.1 backbone with GFP. Lentivirus was produced by transient transfection in 293 T cells. Then MDA-MB-231 and Hs578T cells were infected by ADAM17-shRNA and scramble virus, culture medium was changed after 8–12 h. After 2–3 days, the GFP^+^ cells were FACS analyzed to see the PROCR expression levels.

Sequences of ADAM17-shRNAs are GAAGGTGAATCTAGCTTATTT and CCAGCAGCATTCGGTAAGAAA.

### Statistical analysis

Paired or unpaired two-tailed Student’s t-test was performed when comparing two groups. One-way ANOVA was performed when comparing multiple groups. All the *p*-values were calculated using GraphPad Prism 7 and *p* < 0.05 was considered significant. For all experiments with error bars, the standard error of the mean (S.E.M) was calculated to indicate the variation within each experiment.

## Supplementary Information

**Additional file 1.** Supplementary Fig. S1-S3.

## Abbreviations

CRISPR: Clustered regularly interspaced short palindromic repeat
FACS: fluorescence activated cell sorting
gRNA: guide RNA
TNBC: Triple negative breast cancer
Procr: Protein C receptor
ANOVA: One-way analysis of variance
S.E.M: Standard error of the mean
