# Supplementary material for: Lentiviral CRISPR-guided RNA library screening identified Adam17 as an upstream negative regulator of Procr in mammary epithelium
Source: BMC Biotechnol. 2021 Jul 19;21:42. doi: 10.1186/s12896-021-00703-9 (PMC8290623; doi:10.1186/s12896-021-00703-9)
Supplement: Supplementary file 1 — Additional file 1. Supplementary Fig. S1-S3. [file 12896_2021_703_MOESM1_ESM.pdf]

Wu, et al., Fig S1. Specificity test for real-time qPCR primers.

a

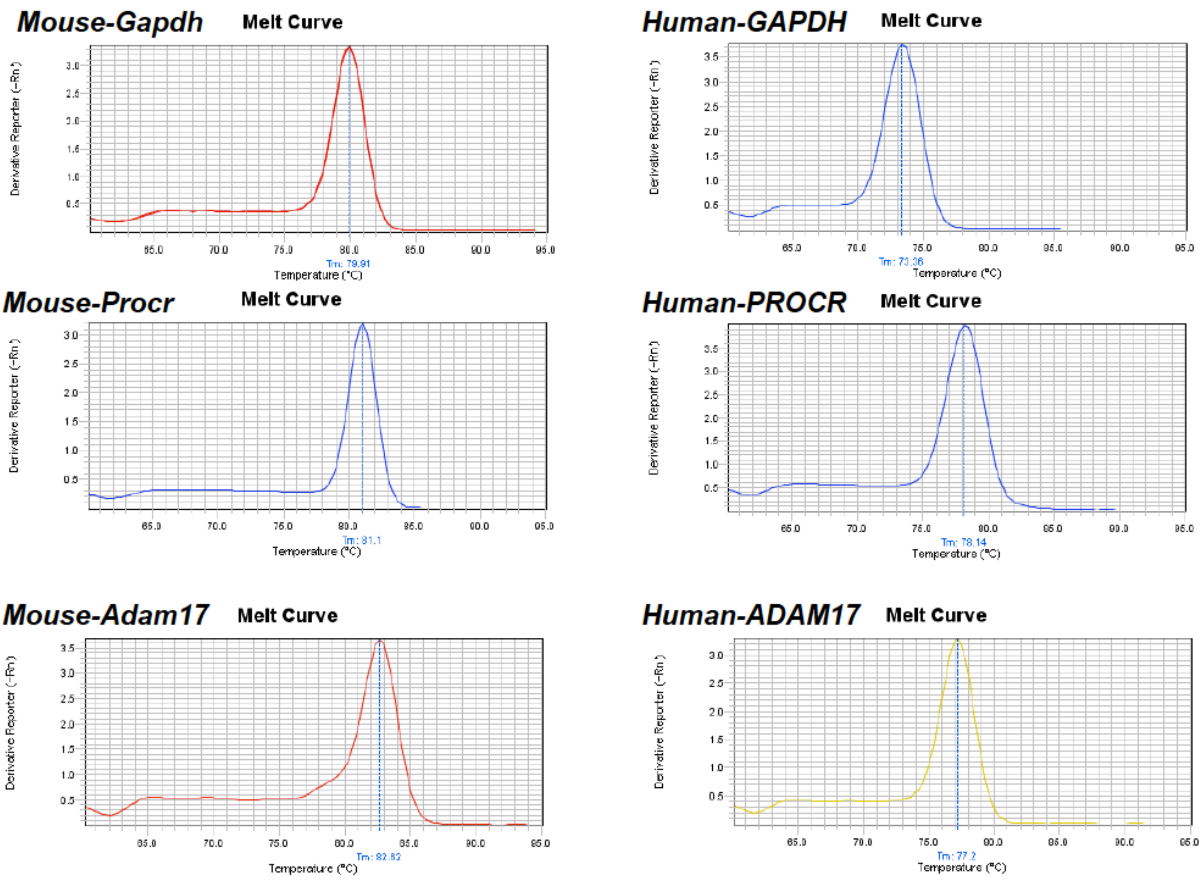

Fig S1. Specificity test for real-time qPCR primers.
(a) Culting curve analysis for real-time qPCR primers.

Wu, et al., Fig S2. Validation of sgRNA efficiency.

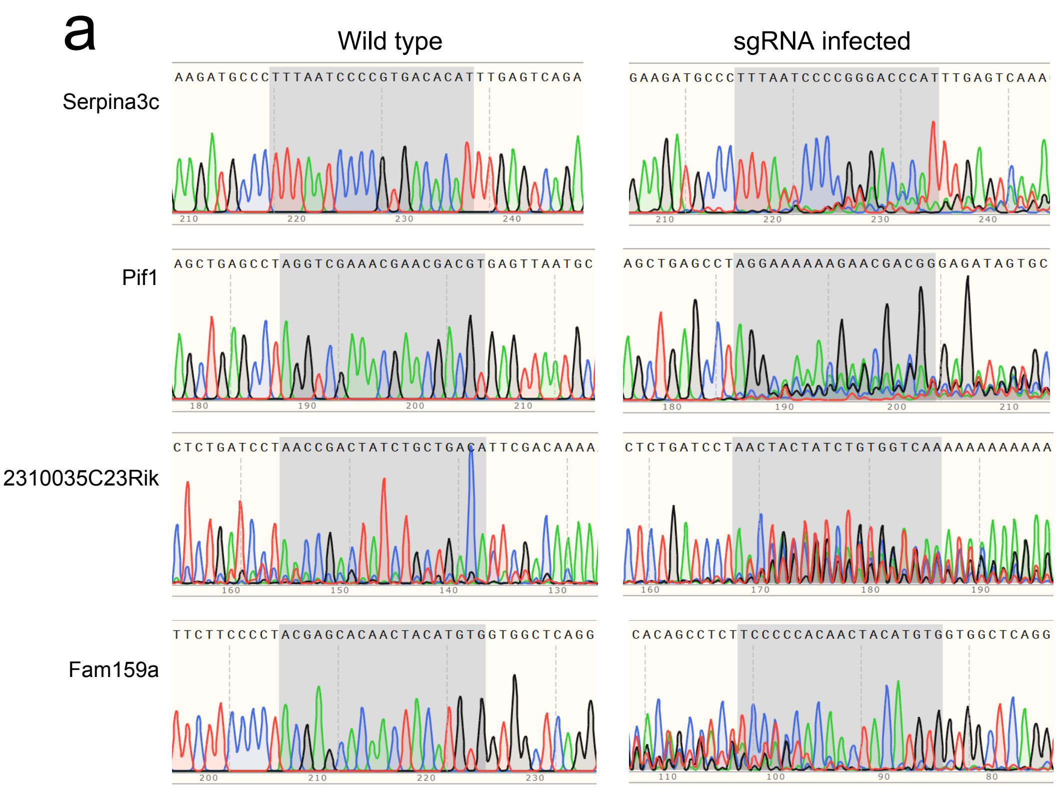

Fig S2. Validation of sgRNA efficiency.

(a) Wild-type and sgRNA infected cells were sequenced to validate the mutantion in *Serpina3c, Pif1, 2310035C23Rik and Fam159a* gene.

Wu, et al., Fig S3. Western Blotting results with different exposure.

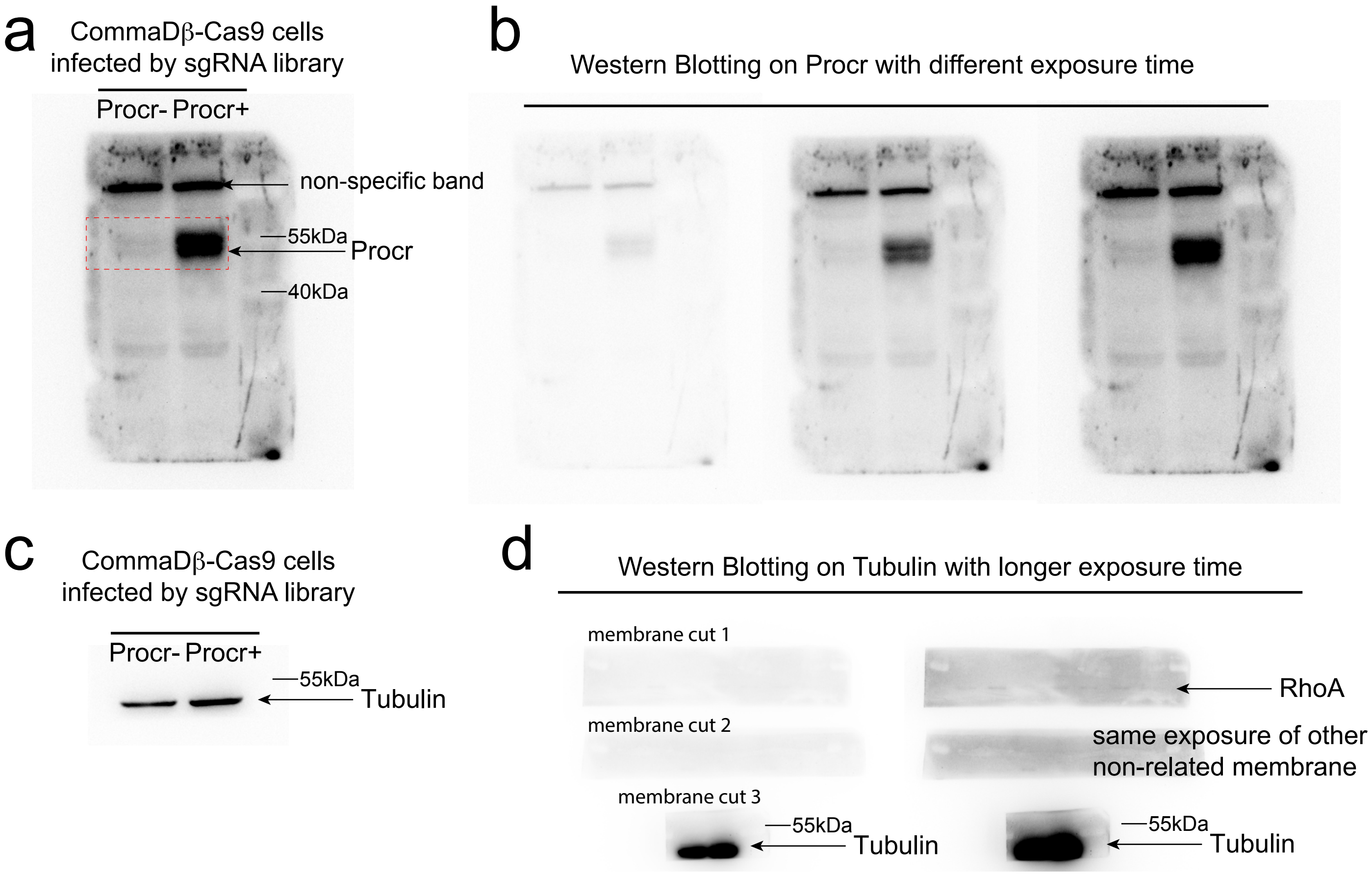

Fig S3. Western Blotting results with different exposure.
(a-b) Western blotting with various exposure time for validating Procr protein levels after sgRNA library infection.
(c-d) Western blotting analysis of Tubulin indicating similar protein loading quantity between samples. (d) Longer exposure of (c) with the presence of other membranes.
